# Mineral composition of repair raspberry (Rubus idaeus L.) fruits

**DOI:** 10.18699/VJGB-22-76

**Published:** 2022-11

**Authors:** S.M. Motyleva, S.N. Evdokimenko, M.A. Podgaetsky, T.A. Tumaeva, Y.V. Burmenko, N.Y. Svistunova, D.V. Panischeva, I.M. Kulikov

**Affiliations:** Federal Horticultural Research Center for Breeding, Agrotechnology and Nursery, Moscow, Russia; Federal Horticultural Research Center for Breeding, Agrotechnology and Nursery, Moscow, Russia; Federal Horticultural Research Center for Breeding, Agrotechnology and Nursery, Moscow, Russia; Federal Horticultural Research Center for Breeding, Agrotechnology and Nursery, Moscow, Russia; Federal Horticultural Research Center for Breeding, Agrotechnology and Nursery, Moscow, Russia; Federal Horticultural Research Center for Breeding, Agrotechnology and Nursery, Moscow, Russia; Federal Horticultural Research Center for Breeding, Agrotechnology and Nursery, Moscow, Russia; Federal Horticultural Research Center for Breeding, Agrotechnology and Nursery, Moscow, Russia

**Keywords:** Rubus idaeus L., cultivars, mineral composition, berries, energy dispersive spectrometry, Rubus idaeus L., сорта, минеральный состав, ягоды, энергодисперсионная спектрометрия

## Abstract

In recent years, raspberry breeding has shifted its emphasis from agronomic performance to characteristics related to the sensory qualities of the fruit and its potential health benef its. The therapeutic and preventive properties of raspberries are related to their biochemical composition. In this regard, the purpose of the work was to determine the content of macro- and micronutrients in fruits of different cultivars of repair raspberry using modern high-tech analytical methods and the selection of genetic sources of the analyzed elements for further breeding. The objects of the research were 17 cultivars of repair raspberry of different ecological and geographical origin from the genetic plant bioresource collection of FSBSO ARHCBAN. It was found that the ash residue of berries contains 12 major elements, which form the following descending series: K > P > Mg ≥ Mo > Ca > S ≥ Ni > Zn > Mn > Se > Fe ≥ Co. The largest proportion of ash residue in raspberry fruits is K. Depending on the cultivar, its quantity averaged from 12.81 wt % (Samorodok and Karamelka) to 22.37 wt % (Atlant). The minimum K content was observed in the ash of the Carolina cultivar (5.62 wt %), while in berries of this cultivar Mg (2.91), Ca (2.62) and Zn (0.14 wt %) accumulated above average. Among the group of early maturing cultivars, the cultivar Yubileinaya Kulikova stands out with a high content of Mo (4.63), Ca (2.19), Fe (0.25) and Co (0.21 wt %). The cultivar Pingvin is characterized by a high content of K (22.65) and Se (0.31 wt %). The medium maturity cultivar Samorodok is characterized by a higher content of P (4.08), S (0.47), Ni (0.51) and Zn (0.26 wt %). Among the late maturing cultivars, the cultivar Poranna Rosa stands out with the preferential accumulation of nine elements: Mg (2.98), P (4.42), S (0.36), K (20.34), Ca (1.71), Mn (0.14), Co (0.13), Se (0.21) and Mo (3.08 wt %). Correlation relationships between the elements have been established. Samples with the highest accumulation of macro- and microelements in berries represent genetic sources for further selection of raspberry for improvement of the mineral composition of fruits.

## Abstract (ru)

В последние годы акцент в селекции малины сместился с агрономических показателей на характеристики, связанные с сенсорными качествами плодов и потенциальной пользой их для здоровья. Терапевтические и профилактические свойства малины обусловлены ее биохимическим составом. В связи с этим целью работы было определение содержания макро- и микроэлементов в плодах различных сортов ремонтантной малины с помощью современных высокотехнологичных аналитических методов и выделение генетических источников анализируемых элементов для дальнейшей селекции. Объектом исследований служили 17 сортов малины ремонтантного типа различного эколого-географического происхождения из генетической биоресурсной коллекции растений ФНЦ Садоводства. Установлено, что в зольном остатке ягод содержатся 12 основных элементов, которые образуют убывающий ряд: K > P > Mg ≥ Mo > Cа > S ≥ Ni > Zn > Mn > Se > Fe ≥ Co. Наибольшую долю зольного остатка в плодах малины составляет K. В зависимости от сорта его количество в среднем изменяется от 12.81 мас.% (Самородок и Карамелька) до 22.37 мас.% (Атлант). Минимальное содержание K отмечено в золе сорта Carolina (5.62 мас.%), при этом в ягодах этого сорта выше средних значений накапливаются Mg (2.91), Са (2.62) и Zn (0.14 мас.%). Среди группы сортов раннего срока созревания высоким содержанием Mo (4.63), Cа (2.19), Fe (0.25) и Co (0.21 мас.%) выделяется сорт Юбилейная Куликова. Сорт Пингвин характеризуется высоким содержанием K (22.65) и Se (0.31 мас.%). Сорт Самородок среднего срока созревания отличается повышенным содержанием Р (4.08), S (0.47), Ni (0.51) и Zn (0.26 мас.%). Среди сортов позднего срока созревания по преимущественному накоплению девяти элементов выделяется сорт Poranna Rosa: Mg (2.98), P (4.42), S (0.36), K (20.34), Cа (1.71), Mn (0.14), Co (0.13), Se (0.21) и Mo (3.08 мас.%). Установлены корреляционные связи между элементами. Образцы с наибольшим накоплением макро- и микроэлементов в ягодах являются генетическими источниками для дальнейшей селекции малины на улучшение минерального состава плодов.

## Introduction

Raspberries are one of the most popular berry crops in household farms and industrial production. In recent years, raspberry selection has shifted the focus from agronomic characteristics to characteristics related to sensory qualities of the fruit (Jennings et al., 2016) and potential health benefits (Mazzoni et al., 2016). At the same time, significant advances were made in the analytical chemistry of fruits. These new tools generate knowledge that can significantly accelerate the creation of new cultivars that meet consumer expectations in terms of sensory perception and the health benefits of eating fruit. In recent years, significant researches have identified environmental, biochemical, and genetic factors underlying the accumulation of certain compounds in raspberry fruits (Kowalenko, 2005; Dresler et al., 2015).

Raspberries are a source of biologically active compounds and minerals that have a positive effect on human health (Pereira et al., 2018; Eremeeva et al., 2019). Minerals belong to the vital components of nutrition (micronutrients) with a wide variety of physiological functions. They play an important role in plastic processes, the formation and construction of body tissues, in particular, the bones of the skeleton. Mineral substances are necessary for maintaining acid-base balance in the body, creating a certain concentration of hydrogen ions in tissues and cells, interstitial and intercellular fluids, as well as giving them osmotic properties that ensure the normal course of metabolism. Mineral elements have antioxidant properties, are involved in redox processes, in carbohydrate, protein, vitamin and fat metabolism, in the formation of bone tissue, regulate heat and gas exchange, hematopoiesis, growth, respiration, play an important role in immunobiological reactions, affect water-salt and acid-base balance (Salmanov, Isrigova, 2004; Nile, Park, 2014; Pochitskaya et al., 2017; Makuev et al., 2018). For example, Fe, being an indispensable component of blood, is involved in oxygen transport and oxidative metabolism (Emel’yanova, 2001). Ca is necessary for the formation of bone and connective tissue, is involved in the transmission of nerve impulses and muscle contraction (Erdman et al., 2012). Cu is a part of a number of important enzymes, normalizes cellular metabolism and catalyzes some of the reactions necessary for the normal functioning of the brains and nervous system. Mg is vital for energy metabolism. Mg and Mn are parts of enzymes, are involved in the metabolism of carbohydrates, amino acids and cholesterol (Ferlemi et al., 2016). Zn maintains an optimal concentration of tocopherol, plays an important role in the growth and development of plants, in the formation of the immune response, the function of the nervous system, promotes the absorption of vitamin A (Frassinetti et al., 2006). In the prevention and treatment of age-related diseases, antioxidant strategies based on nutrition are used, including the addition of antioxidants and trace elements in the prevention (Opara, Rockway, 2006).

Significant intervarietal differences in the mineral content of Na, K, Ca, Mg, Fe, Cu and Zn in raspberry fruits of different colors were established by the studies of Akimov et al. (2021). The quantitative and qualitative composition of mineral substances of fruits and berries depends on the botanical species, cultivar, soil and climatic conditions, methods of cultivation, etc. (Nilova et al., 2018). Despite the role of micronutrients, they have not received as much attention as vitamins, and this may be due to the fact that the safety range between deficiency and toxicity of some trace elements is relatively narrow.

Nevertheless, with the spread of knowledge about rational nutrition and the therapeutic and prophylactic properties of fruits and berries among the population, the demand for them, including raspberries, is growing, which is mostly satisfied by repair cultivars (Gambardella et al., 2016; Orzeł et al., 2016; Moreno-Medina et al., 2018; Evdokimenko, 2020). Despite the popularity of repair raspberries in industrial production, there is only fragmentary information about the mineral composition of its fruits in the scientific literature. Comparative studies of the mineral composition of the repair raspberries berries of the Federal Research Center of Horticulture collection have not previously been conducted. Consequently, the systematization of the content of macro- and microelements in the fruits of repair raspberry cultivars using modern high-tech analytical methods and the typification of samples of the Rubus idaeus L. collection is very relevant.

In this regard, the purpose of our work was to determine the content of macro- and microelements in the fruits of various cultivars of repair raspberries using modern high-tech analytical methods and to isolate the genetic sources of the analyzed elements for further selection.

## Materials and methods

The research was conducted in 2021 in the Laboratory of Biochemistry and Physiology of Plants of the Federal State Budgetary Institution of the Federal Research Center of Horticulture. The objects of study were the fruits of 17 repair cultivars of raspberries (Rubus idaeus L.) of various ecological and geographical origin, differing in terms of ripening, color and other economic and biological signs and properties (Table 1). The raspberries were grown on the site of the genetic collection of the Kokinsky experimental station of the Federal Research Center of Horticulture, located (53.154935° N, 34.123027° E), according to the generally accepted technology with late autumn mowing of stems (Kazakov et al., 2016).

**Table 1.**
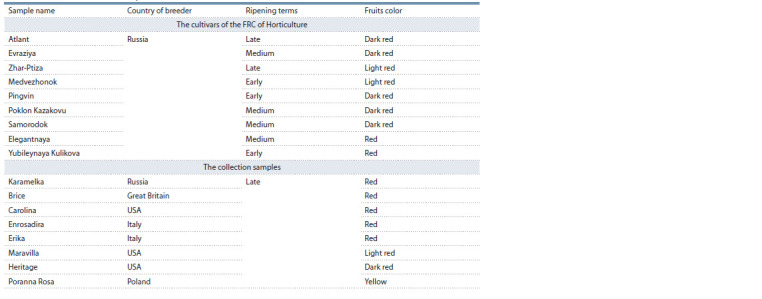
The characteristics of the objects of the research

The soils were gray forest, well cultivated, medium loamy. The depth of the arable layer was 26 cm, the humus content was 3.2 %, Р2О5 was 35 mg per 100 g of soil, K2О was 13.5 mg per 100 g of soil, the reaction of the soil solution was slightly acidic (pH 6.1).

The scheme of planting on the site was single-row, the distance between the rows was 3 m, and between the plants it was 0.5 m. During the season, one spring nitrogen fertilization was carried out (35 kg/ha a.i.). The intervals of the rows in the first half of the growing season were kept under pure steam, and after flowering under natural grassing.

A representative sample of mature raspberries with an average weight of 200 g was dried in a drying oven at a temperature of 50–60 °C. The dried samples were mineralized in a muffle oven Naberterm (Germany) at a temperature of 450 °C in accordance with the Russian State Standard GOST 26929-94 (2002). The resulting ash was dispersed by ultrasound at a frequency of 18 kHz for 15 min. A uniform layer of disperse was applied to a stage table covered with carbon tape.

**Fig. 1.**
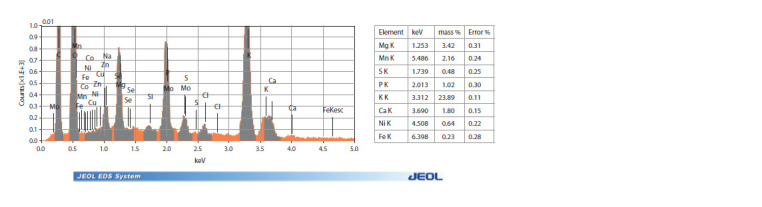
The results of EDS-analysis. Spectral lines of diagnosed elements and a table of results. keV is the energy of the X-ray radiation of the K-level; mass % is the weight part of the element; Error % – detection error recorded by the instrument.

The chemical composition of 12 main ash elements – Mg, P, S, K, Mn, Co, Fe, Ca, Zn, Ni, Se and Mo – was determined by energy dispersion spectrometry (EDS) on an analytical scanning electron microscope JEOL JSM 6090 LA in accordance with the technique (Motyleva, 2018). The resolution of the microscope was 4 nm, the accelerating voltage was 20 kV (image of secondary electrons). The working distance during the elemental analysis was 10 mm. The energy-dispersive microanalysis data were presented in accordance with standard protocols and included images of the microstructures of the sample under study, a table of weight data and spectral lines of the diagnosed elements. An example of an analysis report is shown in Figure 1.

The concentration of the desired elements was determined by the intensity of the spectral lines. The accuracy of chemical analysis was determined as follows: at the concentration of elements from 1 to 5 %, the accuracy was less than 10 %; at the concentration of elements from 5 to 10 %, the accuracy was less than 5 %; and at the concentration of elements more than 10 %, the accuracy was less than 2 %. In total, 10 sites of each sample were examined. The local analysis was 3 mm, and the scanning area was at least 12 μm.

The results were expressed as average values (n = 10) as standard deviation (SD). We used the statistical analysis of the Excel package (Microsoft Excel, v. 2016).

## Results and discussion

Raspberries are known to be rich in minerals (Pereira et al., 2018). 12 main elements that form a descending series have been identified: K > P > Mg ≥ Mo > Ca > S ≥ Ni > Zn > > Mn > Se > Fe ≥ Co. Among the macronutrients, K has had the highest concentration, which is observed in fruits and other berry crops – actinidia, blackberries, strawberries, blueberries (Motyleva et al., 2017; Pereira et al., 2018).

The highest value of K from 20.34 to 22.65 wt % was accumulated in 6 varietal samples – Poranna Rosa, Yubileynaya Kulikova, Zhar-Ptiza, Erika, Atlant and Pingvin. The lowest K content of 5.62 wt % was observed in the fruits of the cultivar Carolina (Table 2). In raspberries of early and late ripening periods, a 3–4 % higher accumulation of K was noted, which may be associated with the genotype of the cultivars

**Table 2.**
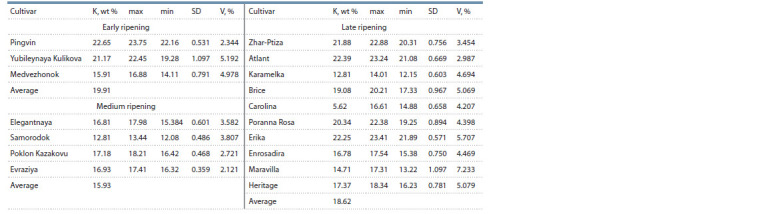
The content of K in the samples of Rubus idaeus L., wt % in ash Notе. Average out of 10 measurements ± SD (standard deviation), V – the coefficient of variation.

Differences in the accumulation of K in raspberries with various fruits colors were revealed. In the berries of darkcolored and red cultivars of raspberries, the average content of K was 18.22 and 15.91 wt %, respectively. In light-colored berries (3 cultivars in total), the content of K ranged from 14.71 (Maravilla) to 21.88 (Zhar-Ptiza) and in yellow-colored berries it was 20.34 (Poranna Rosa) by weight %, respectively. Akimov et al. (2021) also mentioned the high content of K in yellow-colored raspberries of the cultivar Zheltuy Gigant. The content of K in raspberries of domestic and foreign selection cultivars was on average 18.28 and 16.54 wt %, respectively. However, the identified differences in the accumulation of K depending on the color of the berry require further comparative studies on a larger number of cultivars. The variation coefficient of K is low (V = 2.121–5.707 %), which indicates a stable intake of this element in raspberries. In the human body, K is necessary for the work of the heart muscle, maintaining acid-base and water balance. In ionic form, K increases the concentration of other ions and is found in all the organs of the human body (Meathnis et al., 1997).

**Fig. 2.**
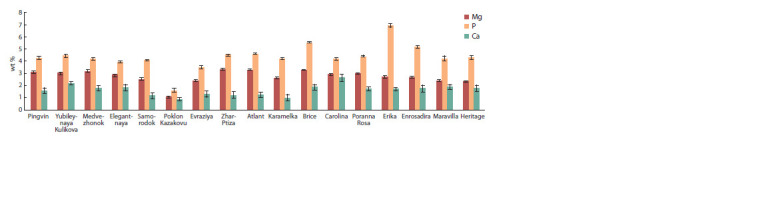
The comparative content of Mg, P and Ca in Rubus idaeus L. berries.

The comparative content of macroelements P, Mg and Ca in raspberries is presented in Figure 2.

The content of P in raspberries varied from 1.59 wt % (Poklon Kazakovu) to 5.19 wt % (Enrosadira). The average content of P in raspberries, depending on the ripening terms, varied within: in berries of early ripening cultivars, its content was 4.29; of medium ripening, 3.27 and of late ripening, 4.81 wt % respectively; the differences were statistically significant at p ≤ 0.05. In berries of foreign selection cultivars, P content is on average 1.5–2.0 % higher than in berries of domestic cultivars. P is involved in many physiological processes, including energy metabolism (in the form of ATP), regulation of acid-base balance, is part of phospholipids, nucleotides, nucleic acids, is necessary for bone mineralization (Avtsyn et al., 1991).

The differences in the content of Mg in raspberries were less expressed than in the content of P – from 1.05 wt % (Poklon Kazakovu) to 3.31 wt % (Zhar-Ptiza). The significant differences in the content of Mg in berries depending on the color of the berries and origin have not been established. In the human body, Mg is a coactor of many enzymes, including energy metabolism, it is involved in protein synthesis and is necessary to support homeostasis (Avtsyn et al., 1991).

Ca ions are involved in blood clotting processes, as well as in ensuring constant osmotic pressure. It is involved in the processes of cell growth and development, it is a part of enzymes and affects metabolism and immunity (Gins et al., 2018). According to Jeong et al. (2008), the main elements in the composition of raspberries are K, P and Ca.

**Fig. 3.**
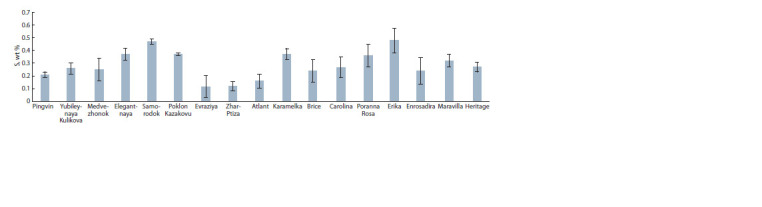
The comparative content of S in the berries of Rubus idaeus L.

The content of S in raspberries ranged from 0.12–0.16 wt % (Evraziya, Zhar-Ptiza and Atlant) to 0.26–0.48 wt % (Yubileynaya Kulikova, Carolina, Erika, Samorodok) (Fig. 3).

**Fig. 4.**
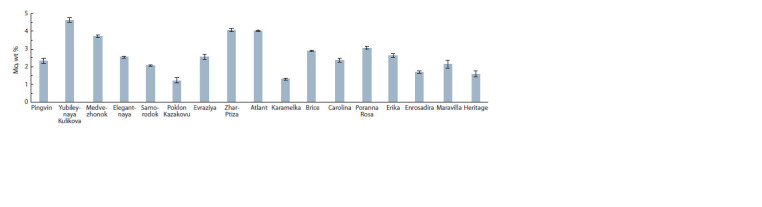
The comparative content of Mo in the berries of Rubus idaeus L.

Raspberries contain a group of trace elements – Mn, Fe, Co, Ni, Zn, Se and Mo. According to the results of our research, Mo in raspberries was contained in the concentrations comparable to Ca and ranged from 1.29 wt % (Poklon Kazakovu and Karamelka) to 4.63 wt % (Yubileynaya Kulikova) (Fig. 4). The high content of Mo was distinguished in the cultivars Atlant and Zhar-Ptiza – 4.01 and 4.07 wt %, respectively.

**Fig. 5.**
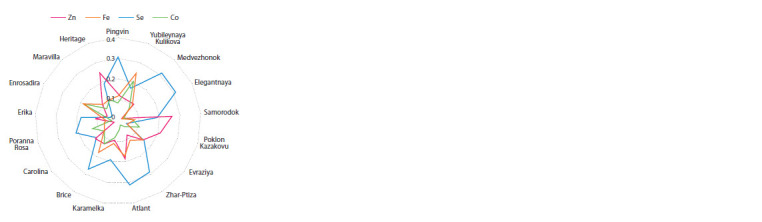
The comparative content of trace elements (Zn, Fe, Se and Co) in the berries of Rubus idaeus L., wt %.

The content of trace elements Zn, Fe, Se and Co in raspberries did not exceed 0.35 wt %. The highest content of Se from 0.27 to 0.31 wt % was found in the berries of the cultivars Brice, Zhar-Ptiza, Atlant and Medvezhonok. The minimum content of this important trace element (0.04 wt %) was noted in the berries of the cultivars Poklon Kazakovu, Enrosadira and Maravilla (Fig. 5). The content of Zn in raspberries ranged from 0.06 (Enrosadira) to 0.25 wt % (Heritage and Samorodok).

The accumulation profiles of Fe and Co in the ash residue of raspberry fruits coincided. The maximum accumulation of these elements was noted in the berries of the cultivar Yubileynaya Kulikova (0.25 and 0.20 wt %) and Enrosadira and Brice (0.18 and 0.19–0.13 wt %). The average content of Fe from 0.11 to 0.15 wt % was found in the berries of the cultivars Karamelka, Maravilla, Heritage and Evraziya.

The proportion of Se in the raspberries of most cultivars was from 0.13 to 0.31 wt %. The maximum content of this trace element was found in raspberries of the cultivars Pingvin, Medvezhonok, Elegantnaya, Atlant, Zhar-Ptiza and Brice. The minimum content of Se (0.4 wt %) was found in the berries of the cultivars Enrosadira, Maravilla and Poklon Kazakovu. Among the cultivars with a high density of berries, the cultivar Atlant stood out, in the ash residue of which the content of K, Mn, Fe, Se and Mo was 1.3, 1.5, 3.8, 1.8 and 1.6 times more than in the berries of other late ripening cultivars. There is evidence that the increase in Se in food in Finland has clearly increased due to the use of fertilizers with the addition of Se (Ekholma et al., 2007).

K, Mg, Ca, Fe, Zn and Mn have been noted as the main elements that are found in red raspberries of the cultivar Willamette (Dragišić Maksimović et al., 2017). There is evidence that Zn and other elements from the group of heavy metals have antimicrobial effect (Daglia et al., 2011). Three key trace minerals, the role of which in antioxidant protection gradually attracts more and more attention, are Zn, Se and Fe. Over the past 20 years, a significant amount of evidence has been accumulated in favor of the role of these elements as cellular antioxidants (Powell, 2000). One of the ways in which Zn acts as an antioxidant is the induction of metallothioneins, a group of small molecule amino acid residues, the production of which is induced by Zn in many tissues, including the liver, intestines and kidneys. Metallothioneins have been shown to scavenge free radicals and bind certain oxidants in a relatively inert state and have been shown to act in this way under a variety of conditions, including radiation exposure, drug toxicity, ethanol toxicity, and mutagenesis (DiSilvestro, 2000). Se is an essential element of the antioxidant defense system of the human body, has an immunomodulatory effect, and participates in the regulation of the action of thyroid hormones (Nutrition hygiene…, 2021).

**Fig. 6.**
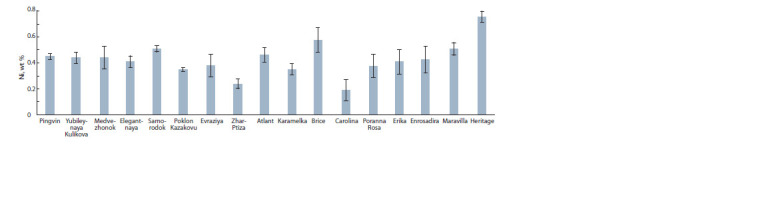
The comparative content of Ni in the berries of Rubus idaeus L.

In the raspberry fruits of all samples, a sufficiently high, slightly varying from the genotype, content of Ni was found, which ranged from 0.35–0.38 wt % (Poklon Kazakovu, Karamelka, Evraziya, Poranna Rosa) to 0.44–0.58 and 0.76 wt % (Pingvin, Yubileynaya Kulikova, Medvezhonok, Brice and Heritage) respectively (Fig. 6). Ni is a transitional element widely distributed in the environment, air, water and soil. Its accumulation can occur from natural sources and anthropogenic activities. Although Ni is ubiquitous in the environment, its functional role as a trace mineral for animals and humans has not yet been recognized. The phytoextraction of Ni depends on the level of the concentration of Ni in the soil (Nordberg et al., 2007; Genchi et al., 2020).

According to the total content of elements in the ash of fruits, the following cultivars were distinguished: Pingvin, Yubileynaya Kulikova, Medvezhonok, Elegantnaya, Zhar-Ptiza, Atlant, Brice, Poranna Rosa, Erika, Enrosadira and Heritage, in the ash residue of which 29–37 of weight % contained the determined elements

The correlation analysis allows to determine the relationship between mineral elements (Table 3). The highest correlation exists between the elements S–Mg (r = 0.9603), Co–S (r = 0.9603), Se–Mg (r = 0.8587) and Co–Ca (r = 0.8577). The average correlation (r = 0.61–0.73) was found between S–P, Mn–Ca, Co–Fe, Se–Ca and S, Mo–S, P and Fe. A low correlation (r = 0.41–0.55) was noted between Ca–S, Mn–S, Fe–S, Ni–Co, Zn–Mn, Fe–Mg and Mo–Mg. There was practically no correlation (r = 0.0085–0.0087) between Se–Ni and Mo–Ca.

**Table 3.**
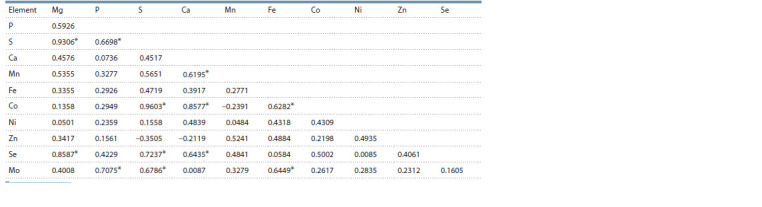
The correlation matrix of mineral (ash) composition of Rubus idaeus L. berries Essential at р <0.05.

## Conclusion

In the fruits of repair raspberries, 12 mineral elements have been identified, the content of which varies depending on the genotype.

The genetic sources of high total accumulation of macroand microelements in the berries are Pingvin, Yubileynaya Kulikova, Medvezhonok, Elegantnaya, Zhar-Ptiza, Atlant, Brice, Poranna Rosa, Erika, Enrosadira and Heritage.

In the selection it is proposed to use the cultivars Medvezhonok, Zhar-Ptiza and Atlant as the sources of increased content of Mg, Mo and Se; the cultivar Yubileynaya Kulikova as the source of accumulation of Ca, Mo and Fe; the cultivars Heritage, Samorodok and Atlant as the source of high content of Zn.

## Conflict of interest

The authors declare no conflict of interest.
